# Identifying Key MicroRNA Signatures for Neurodegenerative Diseases With Machine Learning Methods

**DOI:** 10.3389/fgene.2022.880997

**Published:** 2022-04-21

**Authors:** ZhanDong Li, Wei Guo, ShiJian Ding, Lei Chen, KaiYan Feng, Tao Huang, Yu-Dong Cai

**Affiliations:** ^1^ College of Food Engineering, Jilin Engineering Normal University, Changchun, China; ^2^ Key Laboratory of Stem Cell Biology, Shanghai Jiao Tong University School of Medicine (SJTUSM) and Shanghai Institutes for Biological Sciences (SIBS), Chinese Academy of Sciences (CAS), Shanghai, China; ^3^ School of Life Sciences, Shanghai University, Shanghai, China; ^4^ College of Information Engineering, Shanghai Maritime University, Shanghai, China; ^5^ Department of Computer Science, Guangdong AIB Polytechnic College, Guangzhou, China; ^6^ Bio-Med Big Data Center, CAS Key Laboratory of Computational Biology, Shanghai Institute of Nutrition and Health, University of Chinese Academy of Sciences, Chinese Academy of Sciences, Shanghai, China; ^7^ CAS Key Laboratory of Tissue Microenvironment and Tumor, Shanghai Institute of Nutrition and Health, University of Chinese Academy of Sciences, Chinese Academy of Sciences, Shanghai, China

**Keywords:** neurodegenerative disease, microRNA, feature selection, expression pattern, classification algorithm

## Abstract

Neurodegenerative diseases, including Alzheimer’s disease (AD), Parkinson’s disease, and many other disease types, cause cognitive dysfunctions such as dementia via the progressive loss of structure or function of the body’s neurons. However, the etiology of these diseases remains unknown, and diagnosing less common cognitive disorders such as vascular dementia (VaD) remains a challenge. In this work, we developed a machine-leaning-based technique to distinguish between normal control (NC), AD, VaD, dementia with Lewy bodies, and mild cognitive impairment at the microRNA (miRNA) expression level. First, unnecessary miRNA features in the miRNA expression profiles were removed using the Boruta feature selection method, and the retained feature sets were sorted using minimum redundancy maximum relevance and Monte Carlo feature selection to provide two ranking feature lists. The incremental feature selection method was used to construct a series of feature subsets from these feature lists, and the random forest and PART classifiers were trained on the sample data consisting of these feature subsets. On the basis of the model performance of these classifiers with different number of features, the best feature subsets and classifiers were identified, and the classification rules were retrieved from the optimal PART classifiers. Finally, the link between candidate miRNA features, including hsa-miR-3184-5p, has-miR-6088, and has-miR-4649, and neurodegenerative diseases was confirmed using recently published research, laying the groundwork for more research on miRNAs in neurodegenerative diseases for the diagnosis of cognitive impairment and the understanding of potential pathogenic mechanisms.

## 1 Introduction

Dementia is one kind of cognitive impairment that is characterized by difficulties in memory, language, and behavior. Of all chronic diseases, dementia has become one of the most important contributors to dependence and disability (Iliffe et al., 2009). With an increasing number of morbidity, dementia has become a great concern worldwide (Prince et al., 2016). Unfortunately, there is no cure for this disease at present, and earlier diagnosis and interventions to slow down the disease progress are needed (Iliffe et al., 2009). Therefore, researchers have focused on searching effective diagnostic methods, including the identification of new biomarkers for diagnosis, and interventions for dementia.

Although young-onset cases are increasingly recognized, dementia is typically a condition that affects older people. Alzheimer’s disease (AD) is a progressive neurodegenerative disorder and the most common cause of intellectual deficit in populations older than 65 years. More than 20% of individuals over 80 years of age are affected by AD, and epidemiological data predict that there will be over 35 million AD patients by 2050 (Danborg et al., 2014). Other less common causes of cognitive impairment include vascular dementia (VaD) whose definition and distinction remain controversial, mixed dementia, and dementia with Lewy bodies (DLB) (Mckeith et al., 1996). Diagnosing dementia is markedly difficult due to its insidious onset and diversity of other presenting symptoms such as difficulty in making decisions (Kostopoulou et al., 2008). Recent studies have reported that certain protein biomarkers in cerebrospinal fluid (CSF) can be applied in the clinical diagnosis of AD with a high predictive accuracy (De Meyer et al., 2010). However, such biomarkers have their limitations in differentiating AD from other types of dementia. In addition, biomarkers in CSF require an invasive collection process; thus, new methods through less invasive procedures are needed. Considering that the diagnosis of dementia subtypes is important to manage different therapies, disease courses, and outcomes for different dementias (Robinson et al., 2015), development of better biomarkers for AD and other dementias will contribute to more accurate diagnosis for an early and specialized treatment.

For a better clinical care in disease prevention and treatment, several computational models have been developed to predict dementia risk or subtypes (Stephan et al., 2010). For example, Licher et al. (2019) reported a dementia risk model using optimism-corrected C-statistics, which can be used to identify individuals with high risk of dementia with an accuracy of 0.86. This model was based on comprehensive clinical information such as age, cognitive impairment, and lifestyle factors. Interestingly, a novel machine learning prediction model for dementia risk identification using the voice data from daily conversations was proposed by Shimoda et al. (2021). They applied three strategies including extreme gradient boosting, random forest (RF), and logistic regression methods in developing models, which had AUCs of 0.86, 0.88, and 0.89, respectively. Li et al. (2019) reported a deep learning model for the early prediction of AD using hippocampal magnetic resonance imaging data, which achieved a concordance index of 0.762. In addition, genetic data were taken into account to improve the ability of the prediction model given that many genes were confirmed to be associated with AD (Seshadri et al., 2010). So far, models in dementia prediction lack molecular signatures such as transcriptional expression, which can reflect the underlying pathogenic mechanisms.

MicroRNAs (miRNAs) are small non-coding RNA molecules of approximately 22 nucleotides in length, which have been shown to regulate gene expression by binding to complementary regions of messenger transcripts (Lagos-Quintana et al., 2001). The detection of circulating miRNA levels has been proposed to be a potential diagnostic tool for a number of diseases (Gilad et al., 2008). MiRNAs play a crucial role in the control of neuronal cell development (Mistur et al., 2009). The alteration of the expression of some miRNAs has been shown to relate to various neurological diseases including AD. For example, miR-137, miR-181c, and miR-29a/b were reported to be involved in AD by modulating ceramide levels (Geekiyanage and Chan, 2011). The downregulation of miR-16, miR-195, and miR-103 was observed in the brain of AD patients, and these miRNAs were shown to target the β-site amyloid precursor protein cleaving enzyme 1 (*BACE1*), which is involved in amyloid plaque formation (Bekris et al., 2013). Cogswell et al. found significantly decreased expression of miR-9, which regulates neuronal differentiation, in the human hippocampus of AD patients (Cogswell et al., 2008; Coolen et al., 2013). Different expression patterns of miRNAs have also been found between AD and other neurodegenerative diseases; for example, miR-15a is uniquely elevated in the plasma of AD patients (Bekris et al., 2013). Therefore, miRNAs in the blood or serum are easily accessible and noninvasive biomarkers for diagnosing dementia. In addition, some miRNAs can be used to distinguish different subtypes of dementia for more precise treatment.

In this study, on the basis of the miRNA expression profiles from 1601 serum samples (Shigemizu et al., 2019a), including AD cases, VaD cases, DLB cases, mild cognitive impairment (MCI) cases, and normal controls (NC), we computationally analyzed such expression data. The data was first analyzed by Boruta (Kursa and Rudnicki, 2010), irrelevant miRNA features were excluded. Remaining miRNA features were evaluated by minimum redundancy maximum relevance (mRMR) (Peng et al., 2005) and Monte Carlo feature selection (MCFS) (Dramiński et al., 2007), respectively. Two feature lists were generated, which were fed into incremental feature selection (IFS) (Liu and Setiono, 1998), incorporating random forest (RF) (Breiman, 2001) or PART (Frank and Witten, 1998). As a result, we identified the crucial miRNAs that show the most relevance to the distinction of four different types of dementia and NC, suggesting that these selected miRNAs may play crucial roles in neuronal development. Furthermore, we also identified interesting classification rules, which suggested different miRNA expression patterns on different dementia subtypes and NC. These results can guide further research about the interaction between miRNAs and neurodegenerative diseases. Finally, we constructed two optimal classifiers with high accuracy to group individuals into the corresponding categories (four dementia subtypes and NC). They can be useful tools for the precise diagnosis of dementia subtypes. Our study highlights the potential application of miRNAs in dementia subtype diagnosis, indicating that the prediction framework using serum miRNA expression data can provide feasible therapeutic and diagnostic targets for dementia.

## 2 Materials and Methods

### 2.1 Dataset

In this study, the miRNA expression profiles were obtained from the Gene Expression Omnibus database under the accession code GSE120584 (Shigemizu et al., 2019a; Shigemizu et al., 2019b; Asanomi et al., 2021). These expression profiles include 1,601 samples, which are composed of AD cases, VaD cases, DLB cases, MCI cases, and NC. The sample sizes of different cases are provided in Table 1. A total of 2547 miRNAs were identified in the expression profiles. Subsequently, we performed a computational workflow to detect key miRNA features and expression patterns in the expression profiles.

**TABLE 1:** Sample size for normal control and four neurodegenerative diseases.

Disease case	Sample size
Alzheimer’s disease (AD)	1,021
Vascular dementia (VaD)	91
Dementia with lewy bodies (DLB)	169
Mild cognitive impairment (MCI)	32
Normal control (NC)	288

### 2.2 Boruta Feature Filtering

Aside from the time and energy costs of dealing with a high number of features, most machine learning algorithms work better when the number of predicting features employed is kept as small as possible. We thus applied a Boruta analysis on the miRNA expression profiles to reduce feature dimension and retain important miRNA features (Kursa and Rudnicki, 2010). Boruta is a feature selection approach based on the RF model to access feature importance (Z-score) by comparing the relevance of real features with shadow features, which are randomly shuffled from original features. The python application from https://github.com/scikit-learn-contrib/boruta_py with default parameters was used for Boruta feature selection in this analysis.

### 2.3 Feature Ranking

#### 2.3.1 Minimum Redundancy Maximum Relevance

The mRMR algorithm (Peng et al., 2005) is an entropy-based feature selection method that calculates the mutual information (MI) between a group of features and class variable. The MI is defined as follows:

$$I\left(X;Y\right)=\iint p\left(x,y\right)log\frac{p\left(x,y\right)}{p\left(x\right)p\left(y\right)}dxdy\;$$ (1)

where p(x,y) is the joint probability density function of *X* and *Y*, p(x) and p(y) are the marginal probability density functions of *X* and *Y*, respectively. In the mRMR method, the correlation (*D*) between features and target label and the redundancy (*R*) between features and other features are computed as follows:

$$D=\frac{1}{\left|S\right|}\sum_{x_{i}\in S}I(x_{i};c),$$ (2)

where S is the selected features and $I\left(x_{i};c\right)$ is the MI between feature $x_{i}$ and the target label c .

$$R=\frac{1}{|S{|}^{2}}\sum_{x_{i},x_{j}\in S}I(x_{i},x_{j}),$$ (3)

where $I\left(x_{i},x_{j}\right)$ is the MI between feature $x_{i}$ and feature $x_{j}$ . To repeatedly add a new feature to a feature subset S , the following objective function is optimized:

maxΦ(D, R), Φ=D−R, (4)

In this study, we used the mRMR program acquired from http://home.penglab.com/proj/mRMR/ to rank all the features obtained by Boruta analysis, resulting in an mRMR feature list.

#### 2.3.2 Monte Carlo Feature Selection

The MCFS method (Dramiński et al., 2007) evaluates the feature importance by creating numerous decision trees. More specifically, for a dataset with *M* features, MCFS first randomly constructs *s* feature subsets with *m* features (*m* << *M*). For each feature subset, *t* decision trees are constructed using the bootstrap sampling method. Finally, *s*✕*t* classification trees are constructed and evaluated. The RI score of feature *g* based on these classification trees is defined as follows:

$$RI_{g}=\sum_{\tau =1}^{st}{\left(wAcc\right)}^{u}\sum_{n_{g}\left(\tau \right)}IG\left(n_{g}\left(\tau \right)\right){\left(\frac{no.\;in\;n_{g}\left(\tau \right)}{no.\;in\;\tau }\right)}^{v}$$ (5)

where wAcc is the weight accuracy of the decision tree τ ; $IG\left(n_{g}\left(\tau \right)\right)$ denotes the gain information of node $n_{g}\left(\tau \right)$ ; $\left(no.in\;n_{g}\left(\tau \right)\right)$ and (no.in τ) represent the number of samples of node $n_{g}\left(\tau \right)$ and the number of samples in tree τ , respectively; and *u* and *v* are parameters that are recommended to be 1. After MCFS processing, all features are ranked in a feature list in descending order of RI values. In this study, we applied the MCFS program developed by Draminski et al., which can be accessed at http://www.ipipan.eu/staff/m.draminski/mcfs.html, for feature sorting, and the parameters were set to default values. The obtained feature list was called MCFS feature list.

### 2.4 Incremental Feature Selection

In the previous analysis, the mRMR and MCFS feature ranking lists were obtained, but it was not possible to determine the optimal feature subsets for classifying disease cases. Thus, the IFS method (Liu and Setiono, 1998) was used in this study to identify the best number of features in a feature list for a specific classification algorithm. IFS first generates a series of feature subsets on the basis of a step size. For example, if the step size equals to 1, the first feature subset includes one top-ranked feature, the second feature subset is made up of two top-ranked features, and so on. Then, the sample datasets represented by these feature subsets are trained by one classification algorithm (RF or PART in this study). The classifiers are evaluated by using 10-fold cross-validation (Kohavi, 1995; Tang and Chen, 2022; Yang and Chen, 2022). The evaluation metrics (e.g., Matthews correlation coefficient [MCC]) for each classifier with different number of features are obtained and used to plot IFS curves, where the X-axis is the number of features and the Y-axis is the evaluation metrics. In the end, the optimal feature subsets that achieves the best classification results are identified, and the optimal classifiers are built.

### 2.5 Classification Algorithms

#### 2.5.1 RF

The RF (Breiman, 2001) is an ensemble learning algorithm that takes decision trees as the base learner. It first produces a number of training sets from the original dataset using a bootstrapping method with randomized put-back sampling. These training sets are then used to train the decision tree model individually, and the generated decision trees are formed into a forest. Lastly, the final result is determined by aggregating the voting results of many tree classifiers. As RF is powerful, it is always an important candidate for constructing efficient classifiers (Chen et al., 2017; Zhao et al., 2018; Chen et al., 2021; Li X. et al., 2022; Li Z. et al., 2022; Chen et al., 2022; Ding et al., 2022). In this study, the RF program in Weka (Frank et al., 2004) was employed with default parameters.

#### 2.5.2 PART

In contrast to black-box models, such as RF, rule learning models may learn rules from data to make discriminations on unknown data, and these rules are commonly expressed in an IF–THEN structure, which clearly expresses the patterns existing in the data. PART is a rule-generating method that combines the Ripper and C4.5 approaches without the need for global optimization (Frank and Witten, 1998). It uses a separate-and-conquer technique to develop several partial decision trees, in which a rule is constructed each time. Then, the instances it covers are eliminated, and rules are created recursively for the remaining instances until the end. The PART program in WEKA was used with the default parameters in this investigation.

### 2.6 SMOTE

The distribution of samples under five cases is uneven, which may lead to the poor performance of the established classifiers. To address this issue, we applied SMOTE methods to increase the sample size of the minority class, which is an oversampling technique presented by Chawla et al. (2002). SMOTE generates synthetic samples randomly between samples of a minority class and their neighbors on the basis of the k-nearest neighbor concept. The SMOTE algorithm in Weka software was used to process the miRNA expression profiles in this investigation, resulting in an equal number of samples in each class. It was necessary to pointed out that SMOTE was only used in evaluating the performance of classifiers in the IFS method. Pseudo samples generated by SMOTE did not participate in the mRMR or MCFS methods as they can influence the feature selection results.

### 2.7 Performance Measurement

For the 10-fold cross-validation, we used the MCC as a predictive metric for the evaluation of classifiers. In this study, considering that the analyzed miRNA dataset includes multiple disease cases, the multi-categorical version of MCC (Gorodkin, 2004) was applied and calculated as follows:

$$MCC=\;\frac{cov\left(X,Y\right)}{\sqrt{cov\left(X,X\right)cov\left(Y,Y\right)}}$$ (6)

where the binary matrix *X* represents the prediction results, the binary matrix *Y* indicates the real class label, and cov(X,Y) stands for the covariance of the two matrices. The MCC ranges from −1 to 1, with a value closer to 1 indicating stronger model performance.

To fully display the performance of classification models, we also calculated other measurements, including individual accuracy on each class and overall accuracy (ACC). For one class, its individual accuracy was defined as the proportion of correctly predicted samples in this class. The ACC was defined as the proportion of correctly predicted samples.

## 3 Results

### 3.1 Feature Selection Results on miRNA Expression Profiles

**FIGURE 1:**
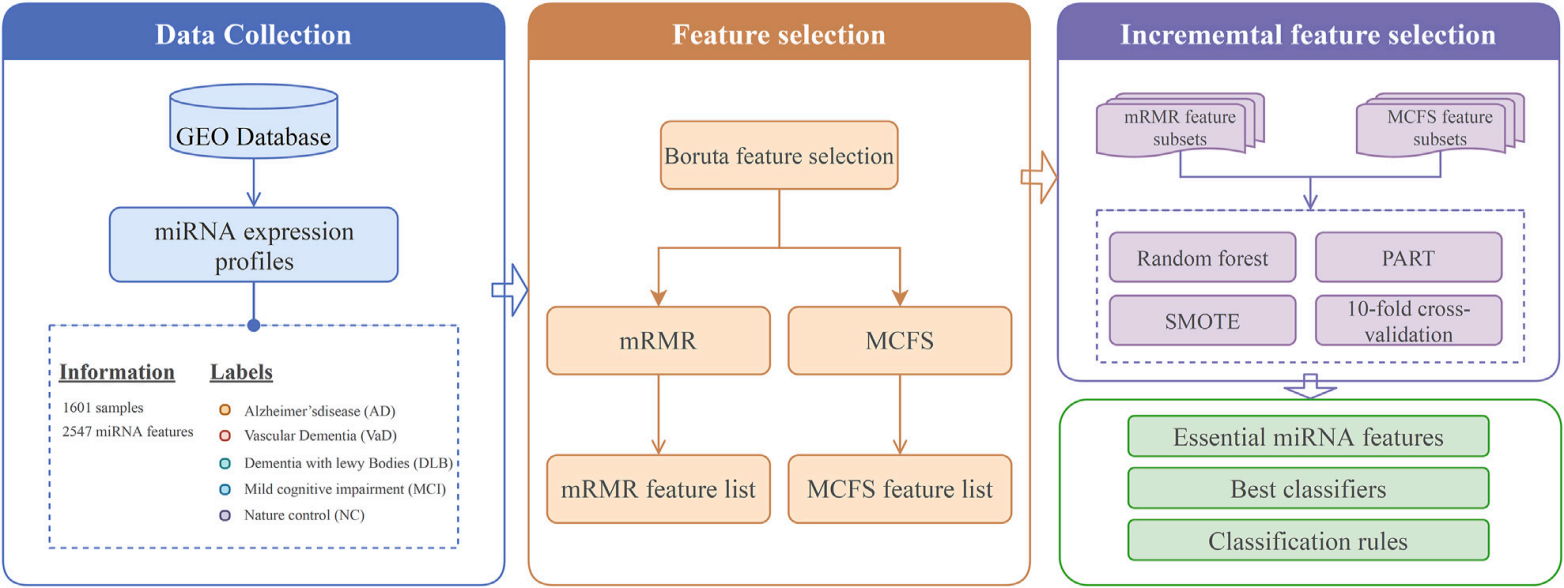
Analysis flowchart for this study, which consists of three main steps: 1) miRNA dataset collection; 2) filtering and ranking of miRNA features in the dataset using Boruta, mRMR, and MCFS; 3) determining the essential miRNA features and building the best classifiers and classification rules using IFS method with RF and PART algorithms.

A flow chart of the present study is illustrated in Figure 1. We started by removing unnecessary features using the Boruta feature selection method, and the 108 retained features are listed in Supplementary Table S1.

**FIGURE 2:**
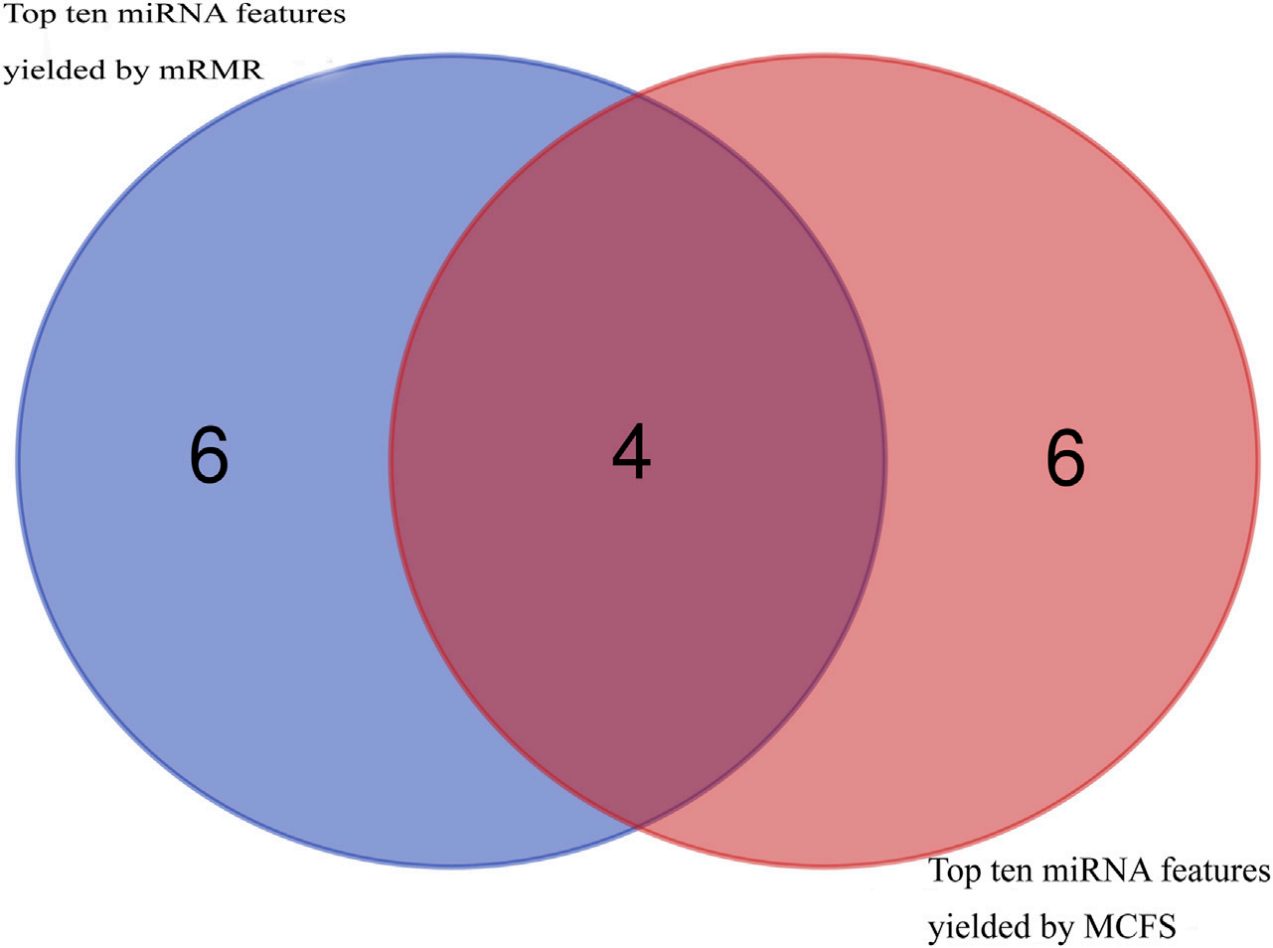
Venn diagram to show top ten miRNA features obtained by mRMR and MCFS methods. Four miRNA features are commonly identified.

Then, using mRMR and MCFS, remaining 108 features were ranked according to feature importance, yielding two ranked feature lists (mRMR feature list and MCFS feature list), as shown in Supplementary Table S1. Top ten miRNA features in these two lists were investigated, as shown in Figure 2. Four miRNAs, including hsa-miR-3184-5p, hsa-miR-1227-5p, hsa-miR-3181, and hsa-miR-6088, appeared in the top 10 features yielded by two methods, highlighting their visibility and importance. The biological roles of these miRNA features will be explored in Section 4.

### 3.2 IFS Results on the mRMR Feature List

**FIGURE 3:**
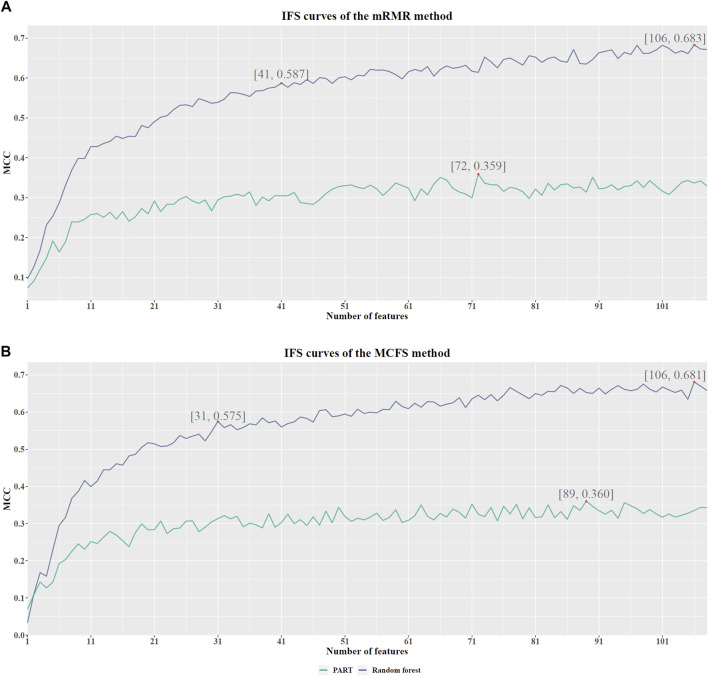
IFS curves with different number of features in RF and PART under the mRMR and MCFS feature lists. **(A)**. With the mRMR feature list, RF reaches the highest point (MCC = 0.683) with the top 106 features, and PART obtains the highest MCC (0.359) when using the top 72 features. The RF with top 41 features also provides high performance (MCC = 0.587). **(B)**. With the MCFS feature list, RF and PART reach the highest points (MCC = 0.681 and 0.360, respectively) at the top 106 and 89 features. The RF with top 31 features also yields high performance (MCC = 0.575).

**FIGURE 4:**
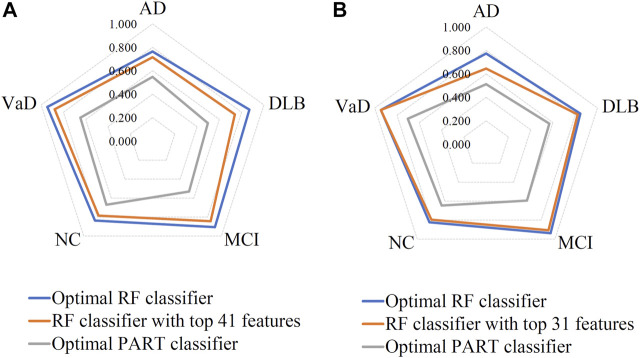
Performance of the key RF and PART classifiers on each class based on mRMR **(A)** and MCFS **(B)** feature lists. AD, VaD, DLB, MCI, and NC stand for Alzheimer’s disease, Vascular dementia, Dementia with Lewy bodies, Mild cognitive impairment and Normal control, respectively.

Based on the mRMR feature list, it was fed into the IFS method with a step size of 1, returning 108 feature subsets. For example, the first feature subset includes the first feature, the second feature subset includes the first two features, and so on. The RF and PART classifiers were trained using the sample set consisting of these feature subsets, and the performance was assessed using 10-fold cross-validation. Obtained measurements are provided in Supplementary Table S2. To clearly display the performance of classifiers on different feature subsets, an IFS curve was plotted for each classification algorithm, which is shown in Figure 3A. When RF was selected as the classification algorithm in the IFS method, the highest MCC was 0.683, which was obtained by using top 106 features. Accordingly, the optimal RF classifier can be built with these features. The ACC of such classifier was 0.802, as listed in Table 2. As for PART, the highest MCC was 0.359. It was obtained by using top 72 features, with which the optimal PART classifier can be built. The ACC of such PART classifier was 0.570, as listed in Table 2. Clearly, the optimal PART classifier was much inferior to the optimal RF classifier. As for their performance on five classes, individual accuracies are shown in Figure 4A. Evidently, the optimal RF classifier provided better performance than the optimal PART classifier on all classes. Both MCI and VaD have an individual accuracy of over 0.900 in the optimal RF classifier.

**TABLE 2:** Performance of key classifiers with different algorithms based on the mRMR feature list.

Classification algorithm	Number of features	ACC	MCC
Random forest	106	0.802	0.683
Random forest	41	0.743	0.587
PART	72	0.570	0.359

Although the optimal RF classifier gave good performance, it was not very proper to do large-scale tests because lots of miRNA features involved. In view of this, we carefully checked the IFS results with RF and found that RF provided the MCC of 0.587 when top 41 features were used (Figure 3A). This classifier yielded the ACC of 0.743 (Table 2). Its performance on five classes is shown in Figure 4A. Although it provided lower performance than the optimal RF classifier, it was much faster as much less miRNA features were needed. This classifier can be an efficient tool to identify four dementia subtypes and NC.

### 3.3 IFS Results on the MCFS Feature List

For the MCFS feature list, the same procedures were conducted. Detailed performance of RF and PART on different number of features is listed in Supplementary Table S3. Likewise, an IFS curve was plotted for each classification algorithm to display the performance of them on different feature subsets, as illustrated in Figure 3B. It can be observed that the highest MCC for RF was 0.681, which was obtained by using top 106 features. Thus, we can build the optimal RF classifier with these features. The ACC of such classifier was 0.803, as listed in Table 3. Its performance on each class is shown in Figure 4B. Compared with the performance of the optimal RF classifier in Section 3.2, their performance was almost equal. As for PART, its highest MCC was 0.360. It was obtained by using top 89 miRNA features. Accordingly, the optimal PART classifier was built using these features. The ACC of this classifier was 0.555 (Table 3). The performance of this classifier on each class is shown in Figure 4B. Evidently, this PART classifier provided equal performance to the optimal PART classifier in Section 3.2. However, they were all inferior to the optimal RF classifiers.

**TABLE 3:** Performance of key classifiers with different algorithms based on the MCFS feature list.

Classification algorithm	Number of features	ACC	MCC
Random forest	106	0.803	0.681
Random forest	31	0.713	0.575
PART	89	0.555	0.360

Similar to the optimal RF classifier in Section 3.2, this optimal RF classifier also need several features. It was necessary to discover another RF classifier with a higher efficiency. After careful checking, we found that RF classifier with top 31 features can produce the MCC of 0.575 (Figure 3B) and ACC of 0.713 (Table 3). Its performance on five classes is shown in Figure 4B. Clearly, it was inferior to the optimal RF classifier. However, it had a higher efficiency because it used much less features. Thus, it can be a useful tool to identify four dementia subtypes and NC. Furthermore, the performance of such RF classifier and RF classifier with top 41 features yielded by mRMR method was almost equal.

### 3.4 miRNA Expression Patterns Extracted From the Optimal PART Classifiers

**FIGURE 5:**
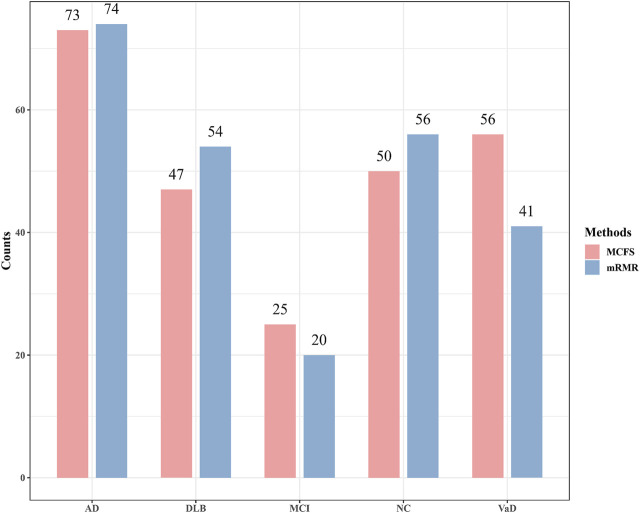
Number of rules generated by the optimal PART classifiers based on mRMR and MCFS feature lists. AD, VaD, DLB, MCI, and NC stand for Alzheimer’s disease, Vascular dementia, Dementia with Lewy bodies, Mild cognitive impairment and Normal control, respectively.

Although the performance of two optimal PART classifier was much lower than two optimal RF classifiers, they can give interpretable rules, which can help us uncover the difference between four dementia subtypes and NC at miRNA level. For the mRMR feature list, the optimal PART classifier used top 72 features. With these features, PART was applied to all samples, resulting in 245 rules. These rules are provided in Supplementary Table S4. Likewise, for the MCFS feature list, top 89 features were adopted in the optimal PART classifier. 251 decision rules were obtained by applying PART on these features, which are also available in Supplementary Table S4. Accordingly, we accessed two groups of decision rules. For each group, each class received some rules. The number of rules for each class on each group is shown in Figure 5. With the exception of MCI, which has a relatively small number of rules, the numbers of rules of other classes were quite considerable. Some key expression rules are listed in Tables 4, 5 and the relevance of these rules in differentiating neurological disorders will be reviewed in Section 4.1.

**TABLE 4:** Some important rules extracted by the optimal PART classifier under the mRMR feature list.

Index	Decision Rules	Class
1	(hsa-miR-6088 ≤ 10.1065) & (hsa-miR-520f-5> 2.0854) & (hsa-miR-6836–3 ≤8.6821) & (hsa-miR-6811–5>1.8782) & (hsa-miR-4667–5> 6.1925) & (hsa-miR-6823–5 > 1.8811) & (hsa-miR-7851–3> 5.1826) & (hsa-miR-4667–5 ≤7.1244) & (hsa-miR-6756–5 ≤8.7714)	Normal control
2	(hsa-miR-6088 ≤ 9.9516) & (hsa-miR-4327 > 7.8591) & (hsa-miR-6861–5> 6.5728) & (hsa-miR-4485–5 ≤6.5037) & (hsa-miR-3622a-3> 4.5067) & (hsa-miR-6875–5 ≤10.0546) & (hsa-miR-7854–3 ≤4.8701)	Alzheimer’s disease
3	(hsa-miR-208a-5> 5.8741) & (hsa-miR-548f-3 ≤2.1097) & (hsa-miR-4667–5> 6.7261) & (hsa-miR-6761–3> 4.7880) & (hsa-miR-520f-5 ≤1.8849)	Vascular dementia
4	(hsa-miR-208a-5> 5.8741) & (hsa-miR-548f-3 ≤2.1097) & (hsa-miR-4649–5>10.8160) & (hsa-miR-3622a-3 ≤4.4907) & (hsa-miR-6070 > 1.8843) & (hsa-miR-663b ≤ 8.7018)	Dementia with lewy bodies
5	(hsa-miR-520f-5 ≤1.8945) & (hsa-miR-6840–3 ≤7.6738) & (hsa-miR-185–5 ≤2.9551)	Mild cognitive impairment

**TABLE 5:** Some important rules extracted by the optimal PART classifier under the MCFS feature list.

Index	Decision rules	Class
1	(hsa-miR-6088 ≤ 10.1065) & (hsa-miR-520f-5> 2.0854) & (hsa-miR-6836–3 ≤8.6821) & (hsa-miR-6811–5> 1.8782) & (hsa-miR-4667–5> 6.1925) & (hsa-miR-4746–3 ≤7.4409) & hsa-miR-3917 > 5.1453) & (hsa-miR-6070 ≤ 2.9233) & (hsa-miR-6869–3>1.8805)	Normal control
2	(hsa-miR-6088 ≤ 9.9516) & (hsa-miR-4327 > 7.8591) & (hsa-miR-1292–3> 4.0332) & (hsa-miR-6861–5> 6.5728) & (hsa-miR-125b-1–3 ≤ 4.7145) & (hsa-miR-128-1-5> 7.0405) & (hsa-miR-7854–3 ≤4.8762) & (hsa-miR-6088 ≤ 9.7663) & (hsa-miR-4506 ≤ 3.6756)	Alzheimer’s disease
3	(hsa-miR-520f-5 ≤1.8945) & (hsa-miR-4485–3> 1.8928) & (hsa-miR-3184–5 ≤8.4938) & (hsa-miR-4496 > 1.8938) & (hsa-miR-6756–5 ≤8.5013) & (hsa-miR-548f-3 ≤1.8935) & (hsa-miR-6822–5> 3.4091) & (hsa-miR-4472 ≤ 6.3202) & (hsa-miR-1914–5 ≤4.1342) & (hsa-miR-6776–3> 4.0568) & (hsa-miR-548o-3> 1.8798)	Vascular dementia
4	(hsa-miR-208a-5> 5.8741) & (hsa-miR-548f-3 ≤2.1097) & (hsa-miR-4667–5 ≤6.7261) & (hsa-miR-4649–5>10.8290) & (hsa-miR-195–3> 1.8967)	Dementia with lewy bodies
5	(hsa-miR-520f-5 ≤1.8945) & (hsa-miR-4485–3> 1.8928) & (hsa-miR-1254 > 6.9170) & (hsa-miR-197–5> 7.3729)	Mild cognitive impairment

### 3.5 Comparison of Optimal Classifiers Without SMOTE

**FIGURE 6:**
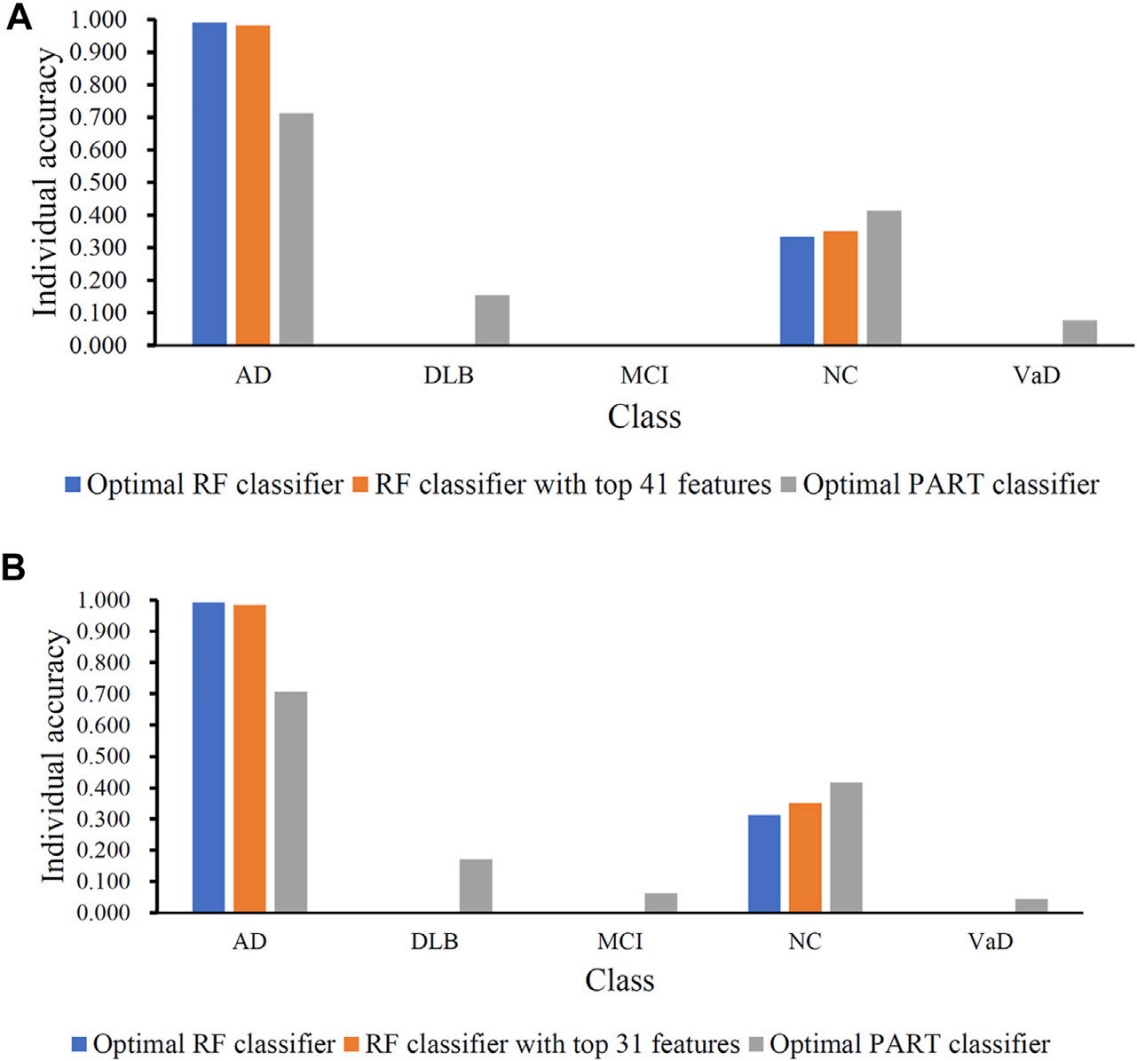
Performance of the key RF and PART classifiers without SMOTE. **(A)**. Classifiers obtained by using mRMR feature list; **(B)**. Classifiers obtained by using MCFS feature list. AD, VaD, DLB, MCI, and NC stand for Alzheimer’s disease, Vascular dementia, Dementia with Lewy bodies, Mild cognitive impairment and Normal control, respectively.

In the IFS method, we employed SMOTE to reduce the influence of imbalanced problem. To elaborate the utility of SMOTE, the RF and PART classifiers mentioned in Sections 3.2, 3.3 were tested when SMOTE was not adopted. All classifiers were assessed by 10-fold cross-validation. The ACCs and MCCs of these classifiers are listed in Table 6. Compared with the ACCs and MCCs listed in Tables 2, 3, MCC greatly decreased by at least 19%, even over 30% for the optimal RF classifiers. The ACC also decreased, but the degree was much smaller than that of the MCC. As the dataset was imbalanced, classifiers directly built on such dataset may be apt to the major classes (AD and NC in this study). Individual accuracies on these classes may be high, whereas individual accuracies on other classes may be low. The individual accuracies shown Figure 6 confirmed this fact. The individual accuracies on AD were very high, followed by those on NC, whereas the individual accuracies on other three classes were very low, even zero. By employing SMOTE, the individual accuracies on AD decreased and those on other classes greatly increased, improving the entire performance of the classifiers. All these indicated the utility of the SMOTE.

**TABLE 6:** Performance of key classifiers without SMOTE.

Feature selection method	Classification algorithm	Number of features	ACC	MCC
mRMR	Random forest	106	0.691	0.323
	Random forest	41	0.690	0.313
	PART	72	0.550	0.158
MCFS	Random forest	106	0.690	0.319
	Random forest	31	0.691	0.317
	PART	89	0.547	0.162

## 4 Discussion

The alteration of miRNA expression has been shown to relate with many pathological processes, including nervous system disorders. In this study, using the expression data of serum miRNAs, two optimal classifiers were constructed with high accuracy to identify the expression features of miRNAs through mRMR and MCFS method. We identified several putative miRNA biomarkers, which displayed strong relevance to the classification, suggesting that these miRNAs have specific effect in different types of neurodegenerative diseases. Additionally, the optimal PART classifiers yielded by mRMR and MCFS feature lists were then applied to generate 245 and 251 decision rules, respectively, which can classify each sample into one of five categories, namely, AD, VaD, DLB, MCI, and NC. In this section, we mainly focused on several optimal and common features identified both by mRMR and MCFS methods, considering that common features are much more important in the classification. We examined the selected features and decision rules and searched for the function and target genes of each miRNA using miRBase, an online database of miRNA sequences and annotation (Kozomara et al., 2018). For some miRNAs that have never been reported, we conducted bioinformatic analysis using miRDB for miRNA target prediction and functional annotation (Liu and Wang, 2019). Through literature review, several pieces of experimental evidence have been found to support the reliability of our prediction.

### 4.1 Analysis of Decision Rules Identified by mRMR and MCFS Methods

The most impactful feature in our computational analysis is miR-3184-5p, the mature miRNA product originating from the stem–loop precursor miRNA through cleavage by ribonuclease. As demonstrated by miRNA array experiment in multiple system atrophy disorders, a downregulated expression of miR-3184-5p was found in the FFPE sample of pons compared with controls, which indicates that this miRNA molecule plays an important role in normal brain development and may contribute in the prevention of neurodegenerative disorders (Wakabayashi et al., 2016). In another research of spinocerebellar ataxia type 3 (SCA3), which is known as a highly heterogeneous neurodegenerative disorder, significantly downregulated expression of miR-3184 was observed in plasma from SCA3 patients compared with healthy controls (Hou et al., 2019). Therefore, we concluded that miR-3184-5p is necessary for the normal function of the brain, and the depletion of this molecule will lead to certain neurodegenerative disorders. Consistent with this finding, several decision rules in which miR-3184-5p is implicated show similar prediction that low expression levels of miR-3184-5p indicate AD and VaD categories, while relatively high expression levels indicate healthy controls.

In many decision rules that indicate the AD category, a relatively high expression of miR-6088 is required for the classification. Although little has been known about this miRNA, we found a report that miR-6088 displays a significantly upregulated expression in patients with stroke compared with NC (Gui et al., 2019). Considering that stroke is a brain disease induced by deficient blood supply and will lead to nervous system injury, we inferred that miR-6088 may also participate in the process of neurodegeneration. Additionally, miR-6088 was identified as one of the differentially methylated genes with high relevance to Parkinson’s disease and neurodegeneration (Marsh et al., 2016), which provides strong support for the crucial role of miR-6088 in pathological processes of the nervous system.

Another important miRNA (miR-4327) is significantly associated with dementia, especially AD, through literature review. In the decision rules, we found that high expression of miR-4327 will lead to the classification of dementia, while relatively low expression indicates the normal cohort. As demonstrated by a miRNA expression profile experiment with Down syndrome, the expression level of miR-4327 was significantly higher in the case group than in the control group, suggesting that dysregulated miR-4327 may be related to abnormal development (Karaca et al., 2018). Individuals with Down syndrome usually show characteristics of damaged brain and intellectual disability, suggesting that miR-4327 affects brain development and results in several pathological processes including neurodegeneration. Moreover, using miRDB website tools, we found that the *OTUD1* gene is predicted as one of the target genes of miR-4327. *OTUD1* encodes a deubiquitinase, and mutations in this gene were reported to be associated with the development of neurological phenotypes including ataxia with cerebellar atrophy and dementia (De Roux et al., 2016). On the basis of this finding, *OTUD1* is necessary for the normal neurological function, while excessive miR-4327 levels may inhibit *OTUD1* transcription and break the normal expression status. Therefore, the high level of miR-4327 is a risk indicator of dementia, which is consistent with our prediction model.

The high expression levels of miR-208a-5p display a strong indication to the categories of dementia in decision rules, suggesting that this miRNA plays a potential role in the associated processes. Several studies have described the role of miR-208a in cardiovascular diseases; for example, circulating levels of miR-208a are significantly elevated in patients with acute coronary syndrome (De Rosa et al., 2011). MiR-208a was undetectable in the blood from healthy individuals, while upregulated expression was observed in the plasma of patients with acute myocardial infarction (Wang et al., 2010). Transgenic overexpression of miR-208a in heart tissue led to hypertrophic growth and arrhythmias in mice (Callis et al., 2009), providing reliable experimental evidence regarding the key function of miR-208a in cardiovascular diseases. Healthy brain functioning is dependent on adequate blood supply, while certain vascular diseases will cause brain injury such as VaD. We inferred that high expression of miR-208a first induces disorders in the vascular system that gradually develop into VaD, which is consistent with the decision rules. Our study is the first to present the role of miR-208a in neurodegenerative diseases, and this will contribute to the clinical diagnosis of dementia.

The high expression of miR-520f, one of the identified features implicated in both decision rules, indicates dementia. MiR-520f was found to be significantly increased in the CSF of patients with Huntington’s disease compared with controls, suggesting that miR-520f can be used as a CSF biomarker for evaluating treatments (Reed et al., 2018). Huntington’s disease is a neurodegenerative disease typically diagnosed in midlife, and this disease shares similar neuropathologic phenotypes to dementia. Thus, we inferred that an elevated level of miR-520f may also influence the pathologic processes of dementia. In addition, miR-520f is also significantly upregulated in multiple system atrophy, and its expression is negatively correlated with the target gene *AKT3* (Kim et al., 2019). *AKT3* has been reported to be related to neuronal insulin resistance in neurodegenerative diseases (Schubert et al., 2004). Taken together, we concluded that miR-520f acts as a transcriptional inhibitor of *AKT3*, and *AKT3* reduction will cause the neuropathologic processes of dementia.

The expression level of miR-1227 can be efficiently used to distinguish the types of dementia and NC in the prediction model, which suggests that miR-1227 is another important functional molecule involved in neurodegeneration. On the basis of a rabbit AD model, the specific expression pattern of miR-1227 was observed, which showed similar profiles to those observed in human AD samples (Liu et al., 2014), indicating the potential role of miR-1227 in AD and other dementia diseases. A recent study reported that *LINC00639*, the target gene of miR-1227, was downregulated in HIV-associated dementia (HAD), a kind of cognitive impairment induced by HIV infection (Li et al., 2018). Even though the pathogenesis of HAD remains unclear, the aberration of certain miRNAs such as miR-1227 can provide novel direction for further research. Similarly, increased expression of miR-1227 was detected in CSF from patients with intracerebral hemorrhage (Shi et al., 2018). In summary, miR-1227 displays distinct expression profiles in many brain injury disorders or dementia, suggesting that it may be an auxiliary diagnostic biomarker for these diseases. These findings confirmed the reliability of our decision rules and implied that the expression criteria of identified miRNAs can be used in disease risk classification and clinical diagnostic.

### 4.2 Analysis of the Top Features Identified by mRMR and MCFS Methods

**FIGURE 7:**
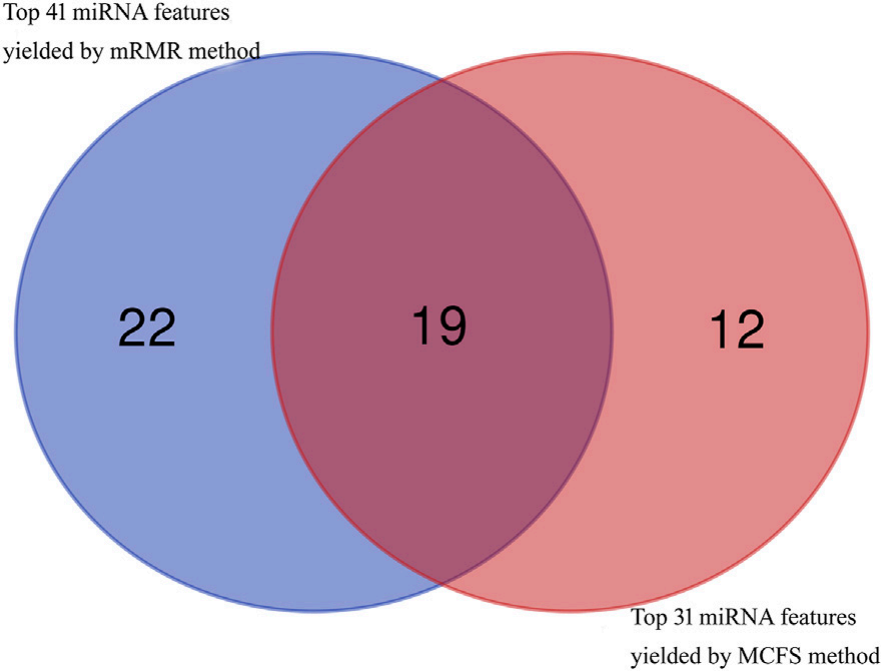
Venn diagram to show top 41 miRNA features obtained by mRMR method and top 31 miRNA features obtained by MCFS method. Nineteen miRNA features are commonly identified.

In addition to the quantitative analysis discussed above, we have also identified many miRNAs that can be used as indicators for dementia. As the RF classifier with less features provided slight lower performance than the corresponding optimal RF classifier, miRNA features used in these two RF classifiers with less features were investigated in this section. Based on the mRMR feature list, 41 miRNA features were obtained, whereas 31 miRNA features were accessed from the MCFS feature list. After taking the union of these two feature subsets, 53 different miRNA features were obtained, which are listed in Supplementary Table S5. A Venn diagram was plotted to show the distribution of these miRNA features in two feature sets, as shown in Figure 7. It can be observed that nineteen miRNA features were commonly identified. These features were thought to be more reliable than others. Some of them were discussed as follows.

MiR-4649-5p exhibits an upregulated expression profile in neurodegenerative disorders (Viswambharan et al., 2017). In amyotrophic lateral sclerosis (ALS), which is a fatal neurodegenerative disease, increasing concentration of miR-4649-5p was observed in the plasma of ALS patients, suggesting that this miRNA can be used in the diagnosis of ALS (Takahashi et al., 2015). On the basis of the miRDB database, we found that miR-4649-5p can target *INSYN2*, a protein coding gene implicated in inhibitory synapses. This synaptic inhibition is fundamental for the functioning of the central nervous system, shaping and orchestrating the flow of information through neuronal networks to generate a precise neural code (Uezu et al., 2016). Therefore, miR-4649-5p plays an important role in neural development, which confirms the reliability of our computational analysis.

MiR-3181 is one of the most related features in our computational analysis, and many studies indicate the close association between this miRNA and vascular diseases. Significantly upregulated miR-3181 was detected in endothelial cells treated with acrolein, which is a component of cigarette smoke and has been implicated in the development of vascular disease, suggesting that this miRNA may improve the diagnosis of vascular disease induced by environmental pollutants (Lee et al., 2015). As discussed previously, the development of vascular disease may be accompanied by brain injury such as VaD, suggesting the role of miR-3181 in dementia. The *TCL1B* gene, which is predicted as one target of miR-3181, showed significant differential expression between Parkinson’s disease patients and NC (Infante et al., 2015). *TCL1B* is also an activator of Akt, a kinase involved in neuron survival (Hashimoto et al., 2013), and abnormal Akt signaling has been reported to induce dopamine neuron degeneration (Greene et al., 2011).

The expression profile of miR-128-1-5p is also a strong indicator for the classification in our analysis. MiR-128 is a neuronally enriched miRNA that plays a crucial role in neuronal differentiation and survival (Guidi et al., 2010). The expression of miR-128 is increased in the hippocampus of AD patients (Lukiw and Pogue, 2007). In addition, upregulated miR-128 can cause a decreased expression of *SNAP25* and lead to the perturbation of neuronal activity (Eletto et al., 2008). These results support the role of miR-128 in neurodegenerative disease. Using RNA sequencing techniques, miR-128 showed decreased expression in Huntington’s disease (Martí et al., 2010). MiR-128 displays distinct expression patterns in different neurodegenerative diseases, indicating its potential capability of distinguishing varied disease subtypes and confirming the ability of our prediction model to classify different dementias.

Besides above commonly identified miRNAs, some miRNAs identified by exact one feature selection method (mRMR or MCFS) were also quite essential. For example, miR-185-5p is identified as one of the most relevant features that contribute to the classification. MiR-185 has been suggested to participate in the pathogenesis of major depression, a psychosocial impairment, and finally lead to suicide. It was thought to influence neuronal and circuit formation by regulating target downstream gene, *TrkB-T1*, which has been associated with suicidal behavior (Serafini et al., 2014). This finding suggests the key role of miR-185-5p involved in nervous system development, physiology, and diseases.

In this section, we discussed the verified or speculative functions of miRNAs identified by our computational analysis. All these miRNAs have been confirmed to contribute to distinguishing patients with dementia from healthy and varied disease subtypes. Strikingly, many miRNAs related to vascular diseases usually play a putative role in neurodegenerative diseases. This finding suggests the interaction between these two distinct disease types. In summary, this study presented a novel computational approach to identify potential biomarkers for diagnosis and therapy, and also set up a basic research foundation for further studies on the detailed pathological mechanism of miRNAs in neurodegenerative diseases.

## 5 Conclusion

We employed a computational analysis approach to discovery key miRNA properties that differentiate normal and neurodegenerative disease subgroups in this work. The Boruta feature selection method was utilized to exclude unnecessary miRNA features, and then mRMR and MCFS were used to rank the remaining features. A series of feature subsets was generated from these ranked feature lists using the IFS method, and the sample data containing these feature subsets was used to train the RF and PART classifiers. As a result, the optimal miRNA biomarker set was identified on the basis of the evaluation metrics of classifiers under varying number of features, and the classification rules were extracted from the optimal PARTs. Finally, the relationship between candidate features including hsa-miR-3184-5p, has-miR-6088, and has-miR-4649 and neurodegenerative diseases was validated in recent studies, confirming the efficacy of our methods and establishing the groundwork for further investigation into the underlying pathogenic mechanisms of miRNAs in neurodegenerative illnesses.

## Data Availability

Publicly available datasets were analyzed in this study. This data can be found here: https://www.ncbi.nlm.nih.gov/geo/query/acc.cgi?acc=GSE120584.

## Supplementary Material

The Supplementary Material for this article can be found online at: https://www.frontiersin.org/articles/10.3389/fgene.2022.880997/full#supplementary-material

Supplementary Table S1 Ranked feature lists analyzed by Boruta feature selection method, mRMR, and MCFS.

Supplementary Table S2 Performance of different classifiers with different numbers of features obtained by IFS method for mRMR feature list.

Supplementary Table S3 Performance of different classifiers with different numbers of features obtained by IFS method for MCFS feature list.

Supplementary Table S4 Rule sets extracted from the optimal PART classifiers based on mRMR and MCFS feature lists.

Supplementary Table S5 Different miRNAs identified by IFS method on mRMR or MCFS feature lists.
